# Morphology and Mechanical Properties of Plantar Fascia in Flexible Flatfoot: A Noninvasive *In Vivo* Study

**DOI:** 10.3389/fbioe.2021.727940

**Published:** 2021-09-15

**Authors:** Zhihui Qian, Zhende Jiang, Jianan Wu, Fei Chang, Jing Liu, Lei Ren, Luquan Ren

**Affiliations:** ^1^ Key Laboratory of Bionic Engineering, Jilin University, Changchun, China; ^2^ Orthopaedic Medical Center, The Second Hospital of Jilin University, Changchun, China; ^3^ School of Mechanical, Aerospace and Civil Engineering, University of Manchester, Manchester, United Kingdom

**Keywords:** flexible flatfoot, plantar fascia, shear wave elastography, morphology properties, mechanical properties

## Abstract

Plantar fascia plays an important role in human foot biomechanics; however, the morphology and mechanical properties of plantar fascia in patients with flexible flatfoot are unknown. In this study, 15 flexible flatfeet were studied, each plantar fascia was divided into 12 positions, and the morphologies and mechanical properties in the 12 positions were measured *in vivo* with B-mode ultrasound and shear wave elastography (SWE). Peak pressures under the first to fifth metatarsal heads (MH) were measured with FreeStep. Statistical analysis included 95% confidence interval, intragroup correlation coefficient (ICC_1,1_), one-way analysis of variance (one-way ANOVA), and least significant difference. The results showed that thickness and Young’s modulus of plantar fascia were the largest at the proximal fascia (PF) and decreased gradually from the proximal end to the distal end. Among the five distal branches (DB) of the fascia, the thickness and Young’s modulus of the second and third DB were larger. The peak pressures were also higher under the second and third MH. This study found a gradient distribution in that the thickness and Young’s modulus gradient decreased from the proximal end to the distal end of plantar fascia in the longitudinal arch of flexible flatfeet. In the transverse arch, the thickness and Young’s modulus under the second and third DB were larger than those under the other three DB in flexible flatfoot, and the peak pressures under the second and third MH were also larger than those under the other three MH in patients with flexible flatfoot. These findings deepen our understanding of the changes of biomechanical properties and may be meaningful for the study of pathological mechanisms and therapy for flexible flatfoot.

## Introduction

Plantar fascia is a ligament that attaches the calcaneus to metatarsals (Orchard, 2012). It plays an important role in passive force transmission (Stecco et al., 2013). Its main task is to stabilize the arch of the foot and reduce the influence of ground reaction force on metatarsal heads (MH) and the longitudinal foot arch (Hicks, 1954; Ker et al., 1987; McKeon et al., 2015). There is a close relationship between plantar fascia and foot function, and studies have shown that when plantar fascia changes, it will produce clinical problems, for example, heel pain (Wearing et al., 2006). Thus, research on plantar fascia has a broad interest.

During the past decades, numerous studies on plantar fascia have been conducted. Guo et al. (2018) found a certain relationship between the mechanical tension of plantar fascia and fiber morphology. Chen et al. (2019a,b) found that people who used forefoot strike were more likely to suffer from plantar fasciitis. Tas and Cetin (2019a) focused on the relationship between plantar pressure distribution and the morphology and mechanical properties of plantar fascia. Welte et al. (2021) revealed the effect of plantar fascia extensibility on the windlass mechanism of plantar fascia. Wang et al. (2019) illustrated the morphology and mechanical properties of plantar fascia in normal feet. These studies strengthen the understanding of the mechanical properties of plantar fascia in normal feet. However, to the author’s knowledge, the morphology and mechanical properties of the whole plantar fascia of flexible flatfeet have not been reported to date.

Flatfoot is a common foot posture abnormality, with the highest incidence of 78% (Sung, 2016), and is characterized by a low medial longitudinal arch (Pehlivan et al., 2009). Flatfoot can be divided into rigid flatfoot and flexible flatfoot. Rigid flatfoot means that the medial longitudinal arch is always missing in both load-bearing and nonload-bearing positions. Flexible flatfoot means that the medial longitudinal arch is missing only in the load-bearing position, while in the nonload-bearing position, it is the same as that of a normal foot (Carr et al., 2016). The abnormal structural changes of the flexible flatfoot under load will gradually lead to changes in the morphology and mechanical properties of the plantar fascia, which may lead to plantar fasciitis and other diseases. The changes in the morphology and mechanical properties of plantar fascia will in turn affect the foot kinematics of patients with flatfoot, resulting in clinical symptoms such as patellar tendinopathy and medial tibial stress syndrome (Kohls-Gatzoulis et al., 2004; Van der Worp et al., 2011; Hamstra-Wright et al., 2015). Studies have shown that the potential cause of plantar fasciitis is the abnormal morphology and mechanical properties of plantar fascia (Wearing et al., 2006; Wu et al., 2011).

Therefore, the objective of this study was to investigate the morphology and mechanical properties of plantar fascia of patients with flexible flatfoot by B-mode ultrasound and shear wave elastography (SWE) *in vivo*. A comprehensive analysis was conducted combined with plantar pressure measurement. The results of the study may provide a meaningful reference and basis for analysis of the pathological mechanism and rehabilitation in patients with flexible flatfoot as well as more accurate definitions for foot finite element models.

## Methods

### Ethics Statement

This study was based on the principles outlined in the Helsinki Declaration, which was approved by the Ethics Committee of the Second Hospital of Jilin University (No. 2020085). All volunteers who participated in the study signed written informed consent agreements.

### Selection of Research Subjects

The subjects of this experiment were patients with flexible flatfeet. They had the typical characteristics in that the medial longitudinal arch was missing only in the load-bearing position, while in the nonload-bearing position, it was the same as that of a normal foot (Carr et al., 2016). An intelligent scanner was employed to confirm the diagnosis and severity of flatfeet, of which the diagnostic principle was the arch index proposed by Cavanagh et al. (1987). The arch index was widely accepted and adopted (Wearing et al., 2004; Wong et al., 2012; Nirenberg et al., 2020; Wang et al., 2020). The inclusion criteria were as follows: 1) healthy male, 20–30 years old; 2) the diagnosis being flexible flatfeet; and 3) no history of other foot diseases. The exclusion criteria were as follows: 1) rigid flatfeet; 2) a history of foot trauma or surgery; 3) presence of systemic diseases that may affect plantar fascia, such as rheumatoid arthritis, diabetes, and gout; and 4) the presence of diseases that affect local plantar fascia, such as calcaneal spur or nodular fasciitis and plantar fibromatosis. Finally, 10 volunteers with 15 flexible flatfeet were included, and the basic characteristics of the volunteers were age, 26.2 ± 1.6 years; weight, 65.2 ± 2.2 kg; height, 175.2 ± 2.7 cm.

### Test Device and Procedure

**FIGURE 1:**
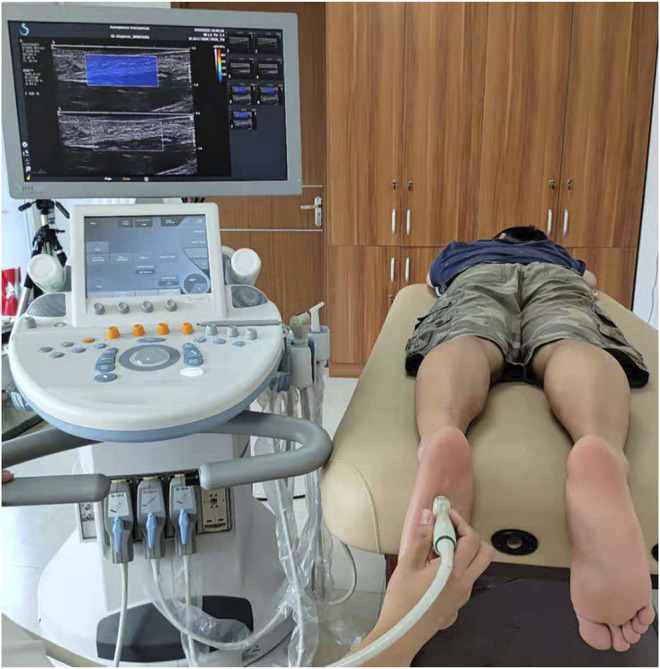
The experimental device and the position of the subjects.

The subjects were asked to avoid intense sports 1 week before the test. B-mode and SWE mode of an Aixplorer ultrasonic scanner (Aixplorer ultrasonic imager, Aix-en-Provence, France) were used to measure the thickness and Young’s modulus of plantar fascia, respectively. The linear transducer frequency was 10–2 MHz for this study. The sampling depth was adjusted according to the positions of plantar fascia. It was set at 1.5–2.5 cm to include the whole plantar fascia, and the mechanical index was 1.0 in this study. During measurement, each subject lay prone on the examination bed, with the lower limbs straight and the feet hanging naturally (Haen et al., 2017) on the edge of the examination bed (Figure 1). The upper body and legs were relaxed.

**FIGURE 2:**
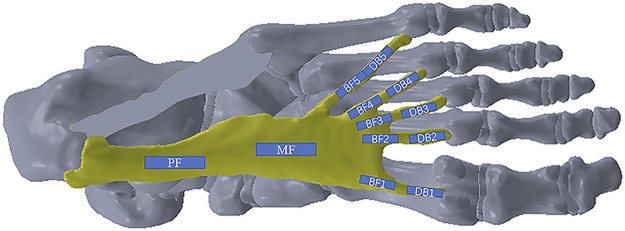
The measuring positions of the plantar fascia.

In order to observe the entire changes in the plantar fascia, it was divided into four main regions: proximal fascia (PF), middle fascia (MF), five branches of fascia (BF1-BF5), and five distal branches (DB1-DB5), 12 positions in total (Figure 2). The PF was measured at a point 1 cm away from the insertion to the calcaneus. The location of the five DBs was defined as the farthest end where the plantar fascia has not been fused with joint capsule. The ultrasonic transducer was parallel to the plantar fascia, and the thickness of plantar fascia was measured in the middle of every position. Subsequently, the elastic measurement via SWE was performed. The width of the square-shaped elastography window (region of interest, ROI) was as large as possible, and the height was set to include the complete plantar fascia. Q-Box^TM^Trace was used to measure Young’s modulus (maximum, minimum, and average, in kPa) of plantar fascia with a length of 1 cm at each position, and Young’s modulus scale was adjusted to 0–600 kPa (Wang et al., 2019). Additionally, the mean Young’s modulus value was used for the data analysis in the study. At each position, Young’s modulus and thickness of plantar fascia were measured three times.

### Plantar Pressure Measurement

FreeStep (Sensor Medica, Italy) was employed to detect the plantar pressure of the subjects during level walking. Subjects were requested to walk normally, without rushing, acceleration, or deceleration. Data were collected barefoot at a self-selected speed (Rao et al., 2011; Hillstrom et al., 2013) along a 2 m walkway, and the walking velocity was 1.33 ± 0.97 m/s. The peak pressures under the first to fifth MH were measured during the push-off stage.

### Statistical Analysis

IBM Statistical Package for the Social Sciences (SPSS) statistical software version 26.0 (SPSS Inc., Chicago, IL, United States) was used to analyze all the data. The 95% confidence interval (95% CI) and intragroup correlation coefficient (ICC_1,1_) were used to measure and evaluate the reliability of plantar fascia thickness and Young’s modulus. Generally, the values of ICC_1,1_ in the ranges of 0–0.40, 0.41–0.6, 0.61–0.79, and 0.8–1.0, respectively, indicate poor, medium, good, and excellent reliability. At the same time, the one-way analysis of variance (one-way ANOVA) was used to compare the differences between different positions of plantar fascia. If the result of one-way ANOVA was *p* < 0.05, least significant difference was used to compare the differences between every two positions of plantar fascia. For least significant difference, *P* values we used had been corrected by the number of pairwise comparisons. Statistical difference was defined as *p* < 0.05. In order to better understand the spatial distribution in thickness and Young’s modulus of plantar fascia, an exponential function (first-order exponential decay) was used to fit and analyze the variation trend of plantar fascia from the calcaneal to the five DB.

## Results

### Intragroup Correlation Results of Thickness and Young’s Modulus

The intragroup correlation results of the thickness and Young’s modulus of the 15 flatfeet are listed in Table 1. The ICC_1,1_ ranged from 0.976 to 0.995 and the corresponding 95% CI was 0.938, 0.998 for thickness of plantar fascia. The ICC_1,1_ ranged from 0.985 to 0.999 and the 95% CI was 0.961, 1.000 for Young’s modulus of plantar fascia.

**TABLE 1:** Intragroup correlation results of thickness and Young’s modulus in 15 flexible flatfeet.

Foot identity	Thickness		Young’s modulus	
	ICC_1,1_ 95%CI	95%CI	ICC_1,1_	95%CI
#1	0.994	(0.984, 0.998)	0.999	(0.998,1.000)
#2	0.991	(0.977, 0.997)	0.995	(0.987,0.999)
#3	0.994	(0.985, 0.998)	0.996	(0.989,0.999)
#4	0.987	(0.964, 0.996)	0.999	(0.996,1.000)
#5	0.981	(0.950, 0.994)	0.998	(0.994,0.999)
#6	0.986	(0.963, 0.996)	0.997	(0.991, 0.999)
#7	0.977	(0.938, 0.993)	0.996	(0.989,0.999)
#8	0.995	(0.986, 0.998)	0.988	(0.968,0.996)
#9	0.988	(0.969, 0.996)	0.992	(0.979,0.998)
#10	0.979	(0.944, 0.993)	0.996	(0.990, 0.999)
#11	0.979	(0.945, 0.993)	0.995	(0.987, 0.999)
#12	0.976	(0.938, 0.993)	0.996	(0.989, 0.999)
#13	0.981	(0.950, 0.994)	0.985	(0.961, 0.995)
#14	0.981	(0.951, 0.994)	0.993	(0.980, 0.998)
#15	0.988	(0.969, 0.996)	0.999	(0.996,1.000)

### Distribution Pattern of Thickness and Young’s Modulus of Plantar Fascia

**FIGURE 3:**
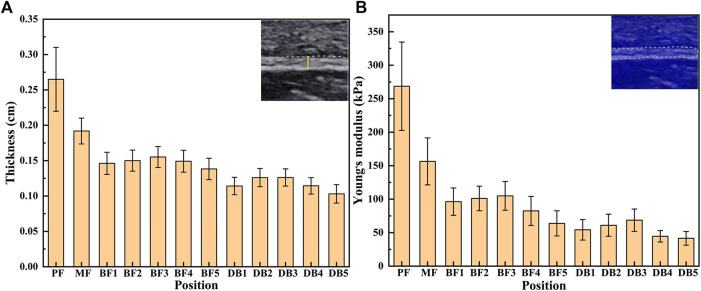
The thickness **(A)** and Young’s modulus **(B)** of plantar fascia of 15 flexible flatfeet.

The results of thickness and Young’s modulus of plantar fascia of 15 flexible flatfeet are shown in Figure 3. The results showed that both the thickness and Young’s modulus of plantar fascia decreased gradually from the proximal end to the distal end. Among the five DB, the thickness and Young’s modulus of the second and third branches were larger than the other three.

The one-way ANOVA was used to compare the differences between different positions of plantar fascia. If the result of one-way ANOVA was *p* < 0.05, least significant difference was used to compare the differences between every two positions of plantar fascia. The one-way ANOVA results showed that the differences in thickness and Young’s modulus between different positions were statistically significant (*p* < 0.05). Least significant difference results showed that, in terms of plantar fascia thickness, PF > MF > all the five BFs > all the five DBs. Among the five DBs, DB2 and DB3 > DB1 and DB4 > DB5. The differences were statistically significant (*p* < 0.05). There was no statistical difference between DB2 and DB3, and there was also no statistical difference between DB1 and DB4 (Table 2). For Young’s modulus, PF > MF > all the five BF > the corresponding position of DB. Among the five DBs, DB2 > DB4 and DB5; DB3 > DB1 and DB4 and DB5; DB1 > DB5. All the differences were statistically significant (*p* < 0.05). There was no statistical difference between DB2 and DB3, and no statistical difference was found between DB2 and DB1. There was also no statistical difference between DB4 and DB5 (Table 3).

**TABLE 2:** *P* value of least significant difference results between different positions in thickness of plantar fascia.

Position/Thickness (mm)	MF	BF1	BF2	BF3	BF4	BF5	DB1	DB2	DB3	DB4	DB5
PF (0.265 ± 0.045)	0.000^a^	0.000^a^	0.000^a^	0.000^a^	0.000^a^	0.000^a^	0.000^a^	0.000^a^	0.000^a^	0.000^a^	0.000^a^
MF (0.192 ± 0.018)	—	0.000^a^	0.000^a^	0.000^a^	0.000^a^	0.000^a^	0.000^a^	0.000^a^	0.000^a^	0.000^a^	0.000^a^
BF1 (0.146 ± 0.016)	0.000^a^	—	0.33	0.024^a^	0.452	0.048^a^	0.000^a^	0.000^a^	0.000^a^	0.000^a^	0.000^a^
BF2 (0.150 ± 0.015)	0.000^a^	0.33	—	0.2	0.824	0.003^a^	0.000^a^	0.000^a^	0.000^a^	0.000^a^	0.000^a^
BF3 (0.155 ± 0.015)	0.000^a^	0.024^a^	0.2	—	0.133	0.000^a^	0.000^a^	0.000^a^	0.000^a^	0.000^a^	0.000^a^
BF4 (0.149 ± 0.015)	0.000^a^	0.452	0.824	0.133	—	0.006^a^	0.000^a^	0.000^a^	0.000^a^	0.000^a^	0.000^a^
BF5 (0.138 ± 0.015)	0.000^a^	0.048^a^	0.003^a^	0.000^a^	0.006^a^	—	0.000^a^	0.002^a^	0.003^a^	0.000^a^	0.000^a^
DB1 (0.114 ± 0.012)	0.000^a^	0.000^a^	0.000^a^	0.000^a^	0.000^a^	0.000^a^	—	0.003^a^	0.003^a^	0.942	0.000^a^
DB2 (0.126 ± 0.013)	0.000^a^	0.000^a^	0.000^a^	0.000^a^	0.000^a^	0.002^a^	0.003^a^	—	0.96	0.004^a^	0.000^a^
DB3 (0.126 ± 0.012)	0.000^a^	0.000^a^	0.000^a^	0.000^a^	0.000^a^	0.003^a^	0.003^a^	0.96	—	0.003^a^	0.000^a^
DB4 (0.114 ± 0.012)	0.000^a^	0.000^a^	0.000^a^	0.000^a^	0.000^a^	0.000^a^	0.942	0.004^a^	0.003^a^	—	0.004^a^
DB5 (0.103 ± 0.013)	0.000^a^	0.000^a^	0.000^a^	0.000^a^	0.000^a^	0.000^a^	0.006^a^	0.000^a^	0.000^a^	0.004^a^	—

a Difference was statistically significant.

*P* values have been corrected (multiplied by k); k represents the number of pairwise comparisons. There were 12 positions; thus, k = 66.

“/” = the same position.

**TABLE 3:** *P* value of least significant difference results between different positions in Young’s modulus of plantar fascia.

Position/Young’s modulus (KPa)	MF	BF1	BF2	BF3	BF4	BF5	DB1	DB2	DB3	DB4	DB5
PF (268.662 ± 65.970)	0.000^a^	0.000^a^	0.000^a^	0.000^a^	0.000^a^	0.000^a^	0.000^a^	0.000^a^	0.000^a^	0.000^a^	0.000^a^
MF (156.407 ± 35.046)	—	0.000^a^	0.000^a^	0.000^a^	0.000^a^	0.000^a^	0.000^a^	0.000^a^	0.000^a^	0.000^a^	0.000^a^
BF1 (96.302 ± 20.356)	0.000^a^	—	0.399	0.126	0.015^a^	0.000^a^	0.000^a^	0.000^a^	0.000^a^	0.000^a^	0.000^a^
BF2 (101.060 ± 18.322)	0.000^a^	0.399	—	0.492	0.001^a^	0.000^a^	0.000^a^	0.000^a^	0.000^a^	0.000^a^	0.000^a^
BF3 (104.938 ± 21.512)	0.000^a^	0.126	0.492	—	0.000^a^	0.000^a^	0.000^a^	0.000^a^	0.000^a^	0.000^a^	0.000^a^
BF4 (82.553 ± 21.637)	0.000^a^	0.015^a^	0.001^a^	0.000^a^	—	0.001^a^	0.000^a^	0.000^a^	0.013^a^	0.000^a^	0.000^a^
BF5 (63.860 ± 18.791)	0.000^a^	0.000^a^	0.000^a^	0.000^a^	0.001^a^	—	0.09	0.613	0.404	0.001^a^	0.000^a^
DB1 (54.271 ± 15.303)	0.000^a^	0.000^a^	0.000^a^	0.000^a^	0.000^a^	0.09	—	0.233	0.011^a^	0.084	0.024^a^
DB2 (61.004 ± 16.479)	0.000^a^	0.000^a^	0.000^a^	0.000^a^	0.000^a^	0.613	0.233	—	0.18	0.004^a^	0.001^a^
DB3 (68.567 ± 16.750)	0.000^a^	0.000^a^	0.000^a^	0.000^a^	0.13	0.404	0.011^a^	0.18	—	0.000^a^	0.000^a^
DB4 (44.500 ± 8.578)	0.000^a^	0.000^a^	0.000^a^	0.000^a^	0.000^a^	0.001^a^	0.084	0.004^a^	0.000^a^	—	0.598
DB5 (41.524 ± 10.270)	0.000^a^	0.000^a^	0.000^a^	0.000^a^	0.000^a^	0.000^a^	0.024^a^	0.001^a^	0.000^a^	0.598	—

a Difference was statistically significant.

*P* values have been corrected (multiplied by k); k represents the number of pairwise comparisons. There were 12 positions; thus, k = 66.

“/” = the same position.

### Peak Pressure Distribution Under Five MHs

**FIGURE 4:**
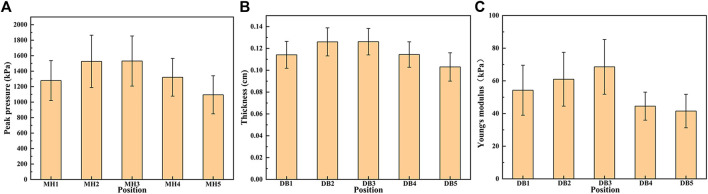
The peak pressure under the five metatarsal heads (MH1–MH5) **(A)**, the thickness **(B),** and Young’s modulus **(C)** in the five distal branches of the plantar fascia in 15 flatfeet.

The peak pressure under five MHs of 15 flexible flatfeet is shown in Figure 4. The pressures under the second and third MH were higher than those under the other three MH, and the differences were statistically significant (*p* < 0.05) (Table 4). This distribution pattern is similar to the thickness and Young’s modulus in the five DBs.

**TABLE 4:** *P* value of least significant difference results between different positions in peak pressure.

Position	MH1	MH2	MH3	MH4	MH5
MH1 (1278.400 ± 258.050)	—	0.000^a^	0.000^a^	0.411	0.000^a^
MH2 (1526.400 ± 338.292)	0.000^a^	—	0.927	0.411	0.000^a^
MH3 (1531.200 ± 323.522)	0.000^a^	0.927	—	0.000^a^	0.000^a^
MH4 (1321.200 ± 243.951)	0.411	0.000^a^	0.000^a^	—	0.000^a^
MH5 (1094.800 ± 246.413)	0.000^a^	0.000^a^	0.000^a^	0.000^a^	—

a Difference was statistically significant.

*P* values have been corrected (multiplied by k); k represents the number of pairwise comparisons. There were five positions; thus, k = 10.

“/” = the same position.

### Spatial Distribution of Plantar Fascia Thickness and Young’s Modulus

In order to better understand the spatial distribution in thickness and Young’s modulus of plantar fascia, an exponential function (first-order exponential decay) was used to fit and analyze the variation trend of plantar fascia from the calcaneal to the five DB.

**FIGURE 5:**
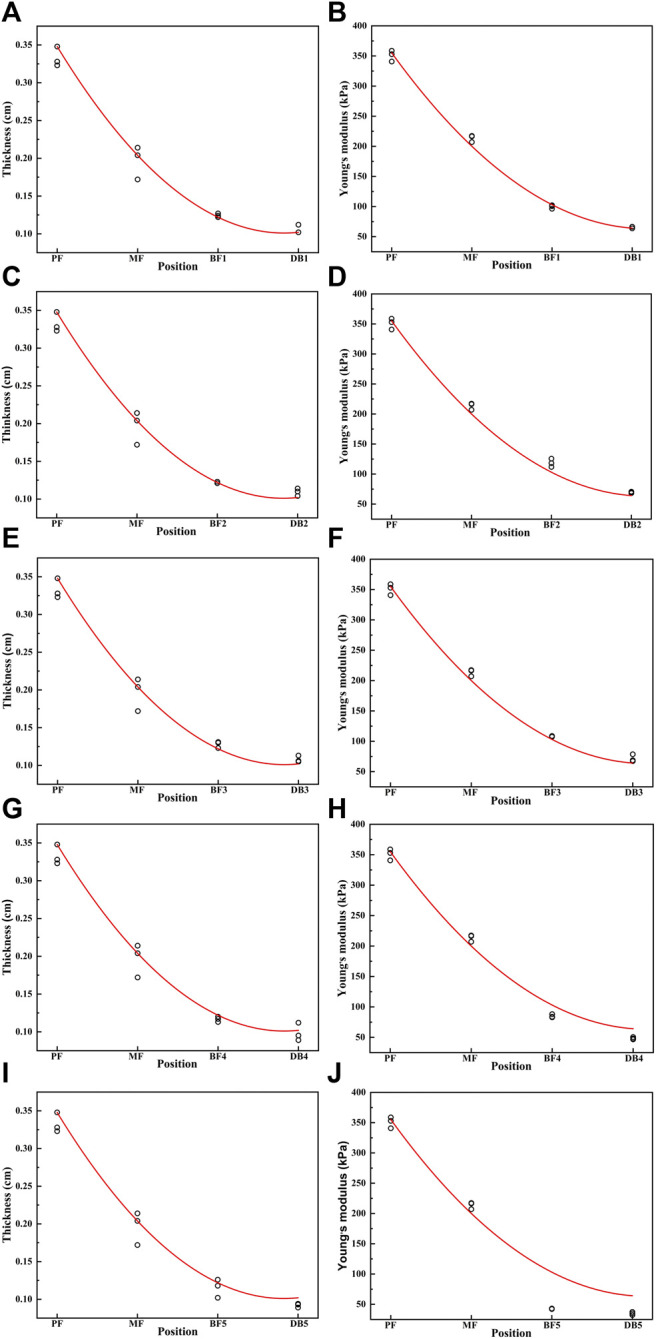
The curve of the thickness and Young’s modulus of the plantar fascia from the calcaneus to the five distal branches in foot #1: **(A)** thickness of the first branch, **(B)** Young’s modulus of the first branch, **(C)** thickness of the second branch, **(D)** Young’s modulus of the second branch, **(E)** thickness of the third branch, **(F)** Young’s modulus of the third branch, **(G)** thickness of the forth branch, **(H)** Young’s modulus of the forth branch, **(I)** thickness of the fifth branch, and **(J)** Young’s modulus of the fifth branch.

**FIGURE 6:**
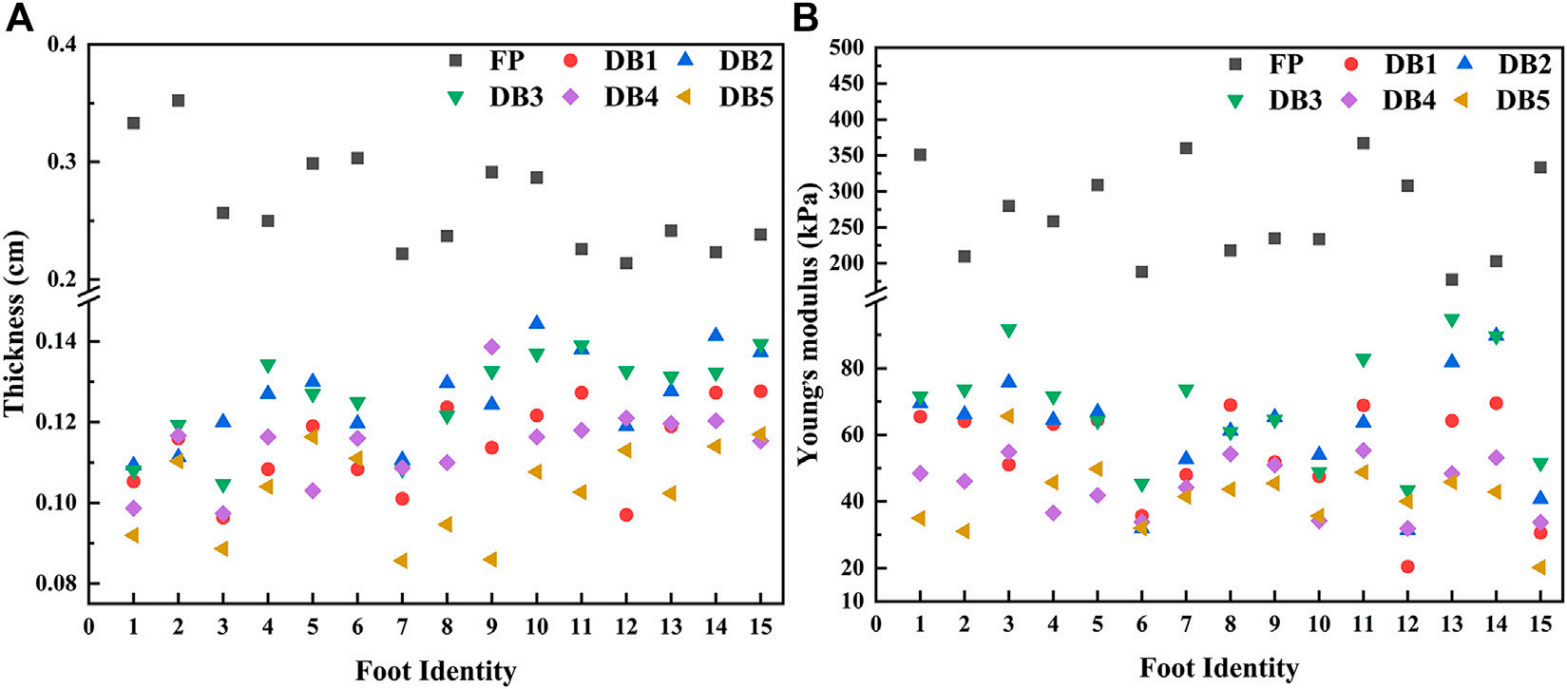
Distribution of plantar fascia thickness **(A)** and Young’s modulus **(B)** at proximal fascia (PF) and the five distal branches in 15 flatfeet.

The spatial distribution of plantar fascia thickness and Young’s modulus of foot #1 is shown in Figure 5. The results showed that the thickness and Young’s modulus of plantar fascia were the largest at the calcaneus tubercle, and the thickness and Young’s modulus of five fascial bundles gradually decreased as plantar fascia extended from the calcaneus to the five toes. The spatial distribution of thickness and Young’s modulus in the other 14 flexible flatfeet also showed a similar tendency. The thickness and Young’s modulus of plantar fascia of 15 flatfeet at PF and five DBs are shown in Figure 6.

## Discussion

This study investigated the morphology and mechanical properties of plantar fascia of patients with flexible flatfoot by B-mode ultrasound and ultrasonic elastography *in vivo*. A comprehensive analysis was conducted combined with plantar pressure measurements.

In order to evaluate the accuracy of the data, the repeatability of the thickness and Young’s modulus data was analyzed in all 15 flexible flatfeet. The results showed that all the values of ICC_1,1_ were more than 0.9, which indicated that the data of the study had good reliability. At the same time, a previous study reported that B-mode ultrasound was a reliable and reproducible method for detecting the thickness of plantar fascia and SWE mode was a reliable and reproducible method for detecting the elasticity of plantar fascia (Wang et al., 2019).

Plantar fasciitis is one of the most common foot musculoskeletal diseases in primary diagnosis and treatment institutions (Thing et al., 2012; Young, 2012), and it is more likely to occur in patients with flatfoot than with normal foot (Riddle et al., 2003). It is characterized by heel pain after rest because it mainly affects the plantar fascia inserted into the calcaneus (Huang et al., 2000). The pain can also extend along the length of the plantar fascia (Thomas et al., 2016; Babatunde et al., 2019). In this study, the maximum Young’s modulus of proximal plantar fascia was 387.1kPa, while that of a normal foot was about 300kPa (Wang et al., 2019). Studies showed that there was a positive correlation between Young’s modulus and tendon force (Yeh et al., 2013; Yeh et al., 2016). Thus, the increased Young's modulus of the proximal plantar fascia indicates that the plantar fascia bears greater stress, leading more easily to the degeneration of plantar fascia (Huffer et al., 2017). The increase of Young’s modulus in plantar fascia near calcaneus attachment in patients with flatfoot may provide a theoretical explanation for the high incidence of plantar fasciitis in patients with flatfoot.

The results of the study showed that the plantar fascia of the flexible flatfoot was spatially dependent from proximal to distal, and the thickness and Young’s modulus of the five branches decreased gradually from proximal to distal. The differences between different parts were statistically significant. This feature of gradient changes is consistent with the results in normal plantar fascia (Wang et al., 2019). In the finite element model, the plantar fascia is often regarded as a linear elastic material, and the whole plantar fascia has the same Young’s modulus (Phan et al., 2021). Thus, the spatial distribution feature (different Young’s modulus in different regions) obtained in this study is helpful to define more accurate material properties for flatfeet finite element models to achieve more meaningful simulation results.

However, among the DB, Wang et al. (2019) showed that the thickness and Young’s modulus between the five branches of the normal plantar fascia were the greatest under the first MH, while this study showed that the thickness and Young’s modulus under the second and third MH were greater in patients with flexible flatfoot. At the same time, this study showed that the peak pressures under the second and third MH were greater than that under the fourth and fifth MH, which was consistent with the results of Buldt et al. (2018) and Hillstrom et al. (2013). It is speculated that this result may be due to the difference in the degree of collapse of the medial and lateral longitudinal arches in patients with flexible flatfoot. These results indicate that, in patients with flexible flatfoot, the degree of collapse of the medial longitudinal arch is more than that of the lateral arch, resulting in higher force and higher pressure on the medial side in the push-off phase. The stronger pressure stimulates plantar fascia, leading to its degeneration (Wearing et al., 2006). Shiotani et al. (2019) also noted that plantar fascia is mechanically stretched, so the morphology and mechanical properties of plantar fascia may be adapted to stress accumulation.

The center of pressure (COP) is defined as the centroid of the pressure distribution at a series of moments in time as the ground reaction is applied over the plantar surface of the foot (Cho and Choi, 2005). It was found that the peak pressures under the second and third metatarsals were higher than those under the other metatarsals. Thus, the COP would move laterally from the first MH. These results were the same as those of Han et al. (2011). They found that, in the normal foot, the trajectory of the COP moved from the lateral heel, moved medially in forefoot, and then ended at the big toe. In flatfeet, the COP moved straight from the heel to the toe without medial shifting in the forefoot. There was a tendency for the COP in flatfoot to shift laterally in the forefoot than the COP in normal foot. These results also confirm our inference; that is, the medial longitudinal arch collapses more than the lateral arch in flatfoot, which leads to the higher force and higher pressure under the second and third metatarsals and the COP moving outward.

Morphologic and mechanical properties of the plantar fascia may be important factors affecting the plantar pressure distribution because the primary task of the plantar fascia is to stabilize the foot arches (McKeon et al., 2015). Studies (Tas and Cetin, 2019a) also show that there is a significant positive correlation between plantar pressure distribution and the thickness of plantar fascia. Higher plantar pressure may lead to plantar fascia hypertrophy. Foot orthoses could modify tissue loading by altering kinematics, kinetics, muscle activity, and sensory feedback (Mills et al., 2010), and they have been demonstrated to have a good therapeutic effect in plantar fasciitis (Buchbinder, 2004). The changes in morphology and mechanical properties of plantar fascia and peak pressure of the forefoot in patients with flexible flatfoot found in our study may provide the basis for the development of new foot orthoses for flexible flatfoot.

There were limitations in this study. Firstly, the sample size was limited to 15 cases, and there was no grading according to mild, moderate, and severe flexible flatfeet. However, the results showed that although the sample size is small and there may be some differences in disease degree among participants, the spatial distribution characteristics of thickness and Young’s modulus of plantar fascia in all 15 flexible flat feet were similar, which indicated that the spatial distribution characteristics are less affected by the disease severity, and the research results may have broad representative significance. Secondly, this study did not include the control group, but our group has previously conducted and published one study on the morphology and mechanical properties of plantar fascia in normal feet (Wang et al., 2019). In addition, the age, height, and body weight of the volunteers who participated in this study are similar to those in our previous published work. Therefore, we cited and employed the published data (normal foot data) as the healthy control group in this study (Wang et al., 2019). Thirdly, though SWE has been used to evaluate the material properties of plantar fascia (Shiotani et al., 2019; Tas and Cetin, 2019b), studies have shown that the shear wave velocity of layered tissue is affected by its thickness and surrounding tissue properties (Helfenstein-Didier et al., 2016; Martin et al., 2018, 2019; Sadeghi and Cortes, 2020); especially when the thickness of the relevant tissue is equal to or less than the wavelength, SWE is no longer applicable (Li et al., 2018). The thickness of plantar fascia measured in this study is millimeter, which is far greater than the wavelength. In addition, the results of Helfenstein-Didier et al. (2016) in measuring the human Achilles tendon show that there is a high correlation between the shear modulus measured by SWE and the new guided wave technology-phase velocity mode, even considering the influence of thickness. Therefore, although the results of the differences between different positions in plantar fascia as well as between patients with flexible flat feet and healthy volunteers in this study may not be affected, it is necessary to explore the influence of thickness on the properties of plantar fascia materials by using guided wave technology in the future.

## Conclusion

This study found a gradient distribution in that the thickness and Young’s modulus gradient decreased from the proximal end to the distal end of plantar fascia in the longitudinal arch of flexible flatfeet. In the transverse arch, the thickness and Young’s modulus under the second and third DB were larger than those under the other three DB in flexible flatfoot, and the peak pressures under the second and third MH were also larger than those under the other three MH in patients with flexible flatfoot. These findings deepen our understanding of the changes of biomechanical properties and may be meaningful for the study of pathological mechanisms and therapy for flexible flatfoot.

## Data Availability

The original contributions presented in the study are included in the article/Supplementary Material; further inquiries can be directed to the corresponding authors.

## Abbreviations

SWE, shear wave elastography; PF, proximal fascia; MF, middle fascia; BF, branches of fascia; DB, distal branches; ROI, region of interest; 95% CI, 95% confidence interval; ICC1,1, intragroup correlation coefficient; one-way ANOVA, one-way analysis of variance; MH, metatarsal head; COP, center of pressure.
